# Digital assessment of banana (*Musa* spp.) genotype resistance to banana weevil (*Cosmopolites sordidus*) compared with expert visual assessment

**DOI:** 10.1371/journal.pone.0352433

**Published:** 2026-06-29

**Authors:** Gerald Mwanje, Jeremy Francis Tusubira, Joyce Nakatumba-Nabende, Reagan Kanaabi, Charles Serebe, Trushar Shah, Rony Swennen, Allan Brown, Gloria Valentine Nakato

**Affiliations:** 1 International Institute of Tropical Agriculture (IITA), Kampala, Uganda; 2 Makerere Artificial Intelligence Lab, Makerere University, Kampala, Uganda; 3 Department of Computer Science, Makerere University, Kampala, Uganda; 4 International Institute of Tropical Agriculture (IITA), Nairobi, Kenya; 5 Department of Biosystems, Katholieke Universiteit Leuven (KU Leuven), Leuven, Belgium; 6 International Institute of Tropical Agriculture (IITA), Arusha, Tanzania; Central Food Technological Research Institute CSIR, INDIA

## Abstract

Accurately assessing weevil damage is critical when evaluating banana germplasm. This enables identification of genotypes resistant to the banana weevil, *Cosmopolites sordidus*, for use as elite parents or for advancement in banana breeding programs. Although visual observation remains the most common phenotyping approach, it is limited by individual bias. This study investigated the potential of image analyses as precise and objective alternatives for assessing weevil damage on the banana corm. ImageJ and four machine learning models, to include YOLO_v11 medium, U-Net 512, SegFormer, and MiT-b0, were explored. 260, 65, and 72 images were used to train, test, and validate the machine learning models, respectively. Phenotyping trials were set up as partially replicated (P-rep) designs with 18 test genotypes and four control genotypes. All plants were produced through tissue culture, raised in pots before infestation with the weevils. At termination, the percentage score of each of the 370 corm samples was evaluated both visually and by image analysis to compare scores across all methods. A significant genotype effect was detected, indicating differences among genotypes in resistance to banana weevils. Furthermore, a significant genotype-by-scoring-method interaction showed that genotype rankings in terms of resistance to banana weevils varied across methods. This emphasized that the choice of scoring approach can affect the magnitude of damage quantified. Visual observation agreed more closely with image analyses for smaller scores and less for larger scores. Results from genotype performance evaluation showed that machine learning methods, except for the YOLO_v11, have a strong level of agreement and can be used interchangeably, giving consistent, reliable, and repeatable measurements. We recommend and have adopted machine learning when scoring weevil damage in the banana corm to avoid individual bias and subjectivity arising from visual observation.

## Introduction

Banana and plantain (*Musa* spp.) are economically important as income and food security crops worldwide. However, their production is constrained by several pests, including the banana weevil, *Cosmopolites sordidus* [1–3]. Banana weevils may cause significant yield losses of up to 40% in the fourth ratoon cycle [4] and 100% beyond the fourth ratoon cycle [5]. Weevil damage from larval feeding causes necrosis of corm tissue, which reduces water and nutrient uptake, decreases bunch size, and weakens plant anchorage, often leading to snapping of the pseudo-stem [2,4].

Banana weevil damage is scored by visual estimation using the naked eye after cutting off the pseudo-stem stump to expose corm tissue, with the observed damage expressed as a percentage of corm damage relative to corm size [5]. The exposed corm tissue is examined for weevil larvae damage by dividing the corm into cross-sections. For each cross-section of the banana corm, the percentage of weevil-damaged tissue is estimated separately for the central cylinder and the outer cortex. These damage scores are then averaged to calculate the total cross-section damage. [6]. Visual observation creates subjectivity that could potentially lead to wrong conclusions by over- or underestimation, especially when scoring many samples. Accurate and reliable data is essential in crop pathology research and, more so in breeding programs as they serve as the basis to discover susceptibility and resistance levels in breeding germplasm [7]. Thus, there is a need to use high-throughput digital phenotyping tools such as ImageJ or other computer-based tools [8]. ImageJ is an open-source Java-based program used in many imaging applications and manipulations to eliminate individual bias and facilitate repeatability and reliability when set ImageJ parameters are kept consistent [9–12]. ImageJ has mostly been used for quantitative measurements of pest and disease damage on plants, as well as for plant growth parameters such as height and width, and canopy cover [13,14]. Recent advances in machine learning have further enhanced these capabilities, allowing for more precise and automated analysis of plant health and growth parameters, thereby improving accuracy and efficiency in agricultural research [15–17].

Digital methods that provide quicker and more accurate quantification of damage by employing computer algorithms [18], can be automated to study a larger number of images at the same time. In addition, they can automatically distinguish infected tissue from healthy tissue as well as visualize small damage that may escape the naked eye [10]. However, the effectiveness of digital data visualization and detection tools heavily depends on the ability to take high-quality images and to use them to train the software to accurately classify and quantify phenotypic variation among test genotype samples. Most digital detection and quantification tools have been tested for vegetative parts of the diseased plants despite symptoms developing in other parts of the plant as well [19,20]. In this study, we explore the efficiency of image analysis using ImageJ and customized machine learning image-based tools in comparison with visual estimation when scoring weevil damage in the banana corm.

## Materials and methods

### Experimental setup

The study was conducted at the International Institute of Tropical Agriculture-Sendusu station in Uganda. One hundred and eighty-five (185) tissue culture-generated plants representing 18 test and four check genotypes, were evaluated in pot trials for resistance to the banana weevil, *Cosmopolites sordidus* (Germar) (Coleoptera: Curculionidae). The test genotypes consisted of the following NARITA banana hybrids (*Musa* spp.): NARITA 1, NARITA 2, NARITA 4, NARITA 7, NARITA 9, NARITA 10, NARITA 11, NARITA 12, NARITA 13, NARITA 14, NARITA 15, NARITA 16, NARITA 17, NARITA 18, NARITA 19, NARITA 21, NARITA 23, and NARITA 26. These hybrids were developed through a joint breeding program between the International Institute of Tropical Agriculture (IITA) and the National Agricultural Research Organisation (NARO) of Uganda. All planting materials were produced via tissue culture at IITA. The experimental design was a partially replicated (P-rep) layout [21], generated and randomized using CycDesigN version 8.0 [22]. It consisted of three blocks, with each test genotype replicated twice with four pseudo-replications per genotype per block (S1 Table). Calcutta 4, a known weevil-resistant genotype [6], and three susceptible landraces to include Mbwazirume (Highland cooking banana), Mchare (Highland cooking banana), and Obino l’Ewai (plantain) were used as checks. All four checks were replicated in all three blocks. 13-Litre plastic buckets, perforated at the base for drainage, were filled with sterilized growth medium consisting of topsoil, farm manure, and sawdust mixed in a 3:1:1 ratio, respectively. Plantlets were grown in the buckets, arranged in the screenhouse following the P-rep design layout, and watered every other day to maintain adequate soil moisture. Adult banana weevils (*Cosmopolites sordidus*) were collected from banana fields using pseudo-stem traps and subsequently reared on corms of susceptible genotype Mbwazirume in buckets for one week to allow acclimatization to screenhouse conditions. Species identity was confirmed based on morphological characteristics using standard taxonomic keys, including a glossy dark brown to black, elongated body (approximately 10–15 mm in length), a prominent curved rostrum, and well-developed elytra with distinct longitudinal grooves. Sex determination for each weevil was conducted based on rostrum punctuation under a stereomicroscope, with males having fully punctuated rostra and females having half or less than half punctuated [23]. Weevils of relatively uniform size were selected for infestation to standardize infestation pressure and reduce experimental heterogeneity in factors such as oviposition rates. At 60 days post-planting, each plant was infested with three female and three male weevils. Following infestation, plants were wrapped with weevil-proof nets to prevent weevil escape (S1 Fig). Plants were watered regularly to maintain moist conditions which are preferred by banana weevils for survival and oviposition, as the pest is highly susceptible to desiccation in dry environments. Data collection was at 60 days post-infestation (dpi) following the cross-section method described in [24]. This study did not require ethical approval as it involved only banana plants and banana weevils, with no human participants, vertebrate animals, or protected species.

### Preparation of corm for image capture and visual scoring

**Fig 1:**
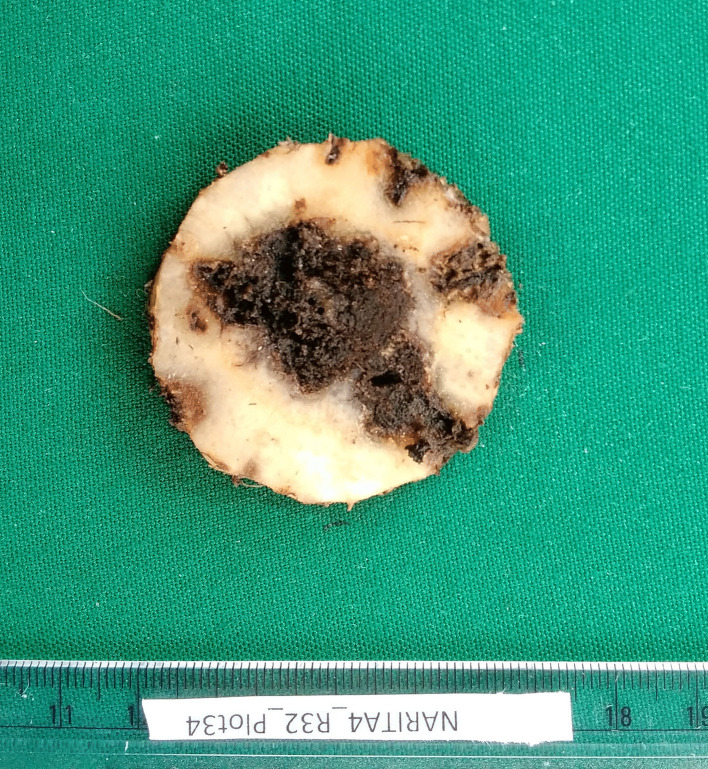
A high-quality 8-megapixel weevil-damaged corm image captured on a green background for image analysis. Brown areas show damage from the banana weevil (0.6X magnification).

The corm was cut transversely, first at the collar region found between the pseudo-stem and corm to expose the upper cross-sectional area, and two centimeters below the collar to expose the lower cross-sectional area. High-quality images (resolution 3264*2448, 8 megapixels, aspect ratio of 4:3) of each cross-section were captured using a Samsung SM-T295 camera (F2.0 aperture, 2.85 mm focal length, 1/100 s exposure). A green background was used during image capture to increase contrast and optimize quality (Fig 1). Genotype name, block, and plot number were also captured. A ruler was incorporated as a reference in all images to ensure precise measurements using ImageJ (Fig 1). The resulting images were saved as Joint Photographic Expert Group (JPEG) format. For each plant, two images representing upper and lower cross-sections were captured, totaling 370 images from 185 plants of 22 genotypes.

Weevil damage was scored visually for the central cylinder and the cortex as described by Gold et al. [5]. The exposed corm area was divided into four quadrants, and visually assessed as a percentage ranging from 0–25% in each quadrant. Total percentage damage in all four quadrants for each cross section was recorded as the weevil damage score for that cross section. This was done for each cross-section to include: (a) outer upper cross-sectional damage (cortex), (b) inner upper cross-sectional damage (central cylinder), (c) outer lower cross-sectional damage (cortex), and (d) inner lower cross-sectional damage (central cylinder). The mean of the four scores was calculated to generate overall damage.

### Image processing using ImageJ

#### Automated background removal for ImageJ-based image analysis.

**Fig 2:**
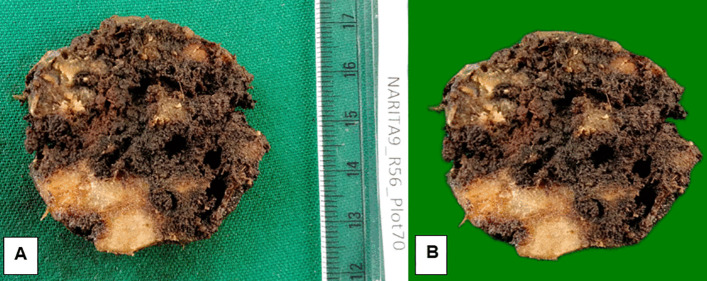
(A)-Original 8-megapixel image of banana corm as taken with the camera; (B)-Banana corm image whose original background has been removed using rembg Python package (0.3x magnification).

Before image analysis, the background was removed using the rembg Python package [25]. Image accuracy was improved by replacing the initial dull green background shown in Fig 2A with a consistent bright green background having an RGB value of 0, 128, 0 as shown in Fig 2B. RGB is an additive color model system representing red, green and blue colors of light used on a digital display screen to reproduce a broad array of colors for sensing and displaying images in electronic systems [26]. The RGB images generated were preserved in their original size and saved as JPEG files with a compression quality of 95, ensuring maximum retention of image quality. Compression quality is the level of perfection in digital images achieved through data minimization without degrading image quality [26].

#### Differentiating and quantifying healthy and damaged regions.

The rembg processed images were imported into the ilastik (version 1.3.0) for pixel labelling to train a custom image classifier to create binary segmentations of healthy and damaged tissue, and differentiate the background from the corm images. We adopted a two-fold training approach that combined manual labeling and automated machine learning algorithms. A subset of imported images was annotated to precisely identify healthy tissue, damaged tissue, and background. These annotations were then used to develop the segmentation framework and generate the labeled dataset.

**Fig 3:**
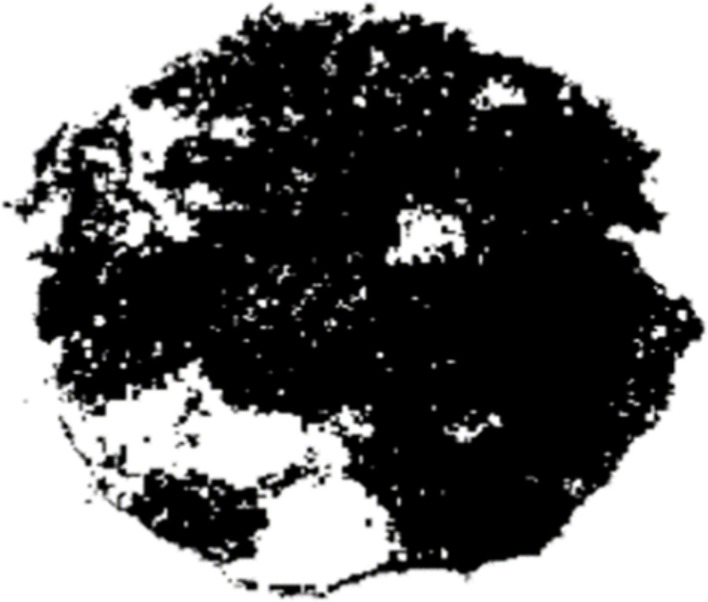
Binary image generated by a trained ilastik machine learning algorithm. Black is the weevil-damaged corm tissue and white the healthy corm tissue (0.4X magnification).

Using the labelled dataset, ilastik was trained to recognize patterns and features characteristic of each tissue class. The trained classifier was then applied to the entire set of rembg-processed images to generate binary masks. In these masks, each pixel was assigned a value of 0 for healthy tissue and 1 for damaged tissue (Fig 3). The trained ilastik model was saved for future analyses.

Particle analysis was performed in ImageJ to quantify healthy and damaged sections in the binary masks. The threshold was set to “Set Threshold (2,2)” for healthy tissue and “Set Threshold (1,1)” for damaged tissue. Quantification was then conducted using ImageJ’s Analyze Particles tool (size: pixels² 0 – infinity, circularity: 0.00–1.00) to obtain area measurements and standard deviation. The percentage of weevil-damaged tissue was calculated as a relative measure by dividing the number of pixels classified as damaged by the total number of pixels representing the corm area, and multiplying the result by 100. This ensured that the results remained consistent and comparable across images, regardless of variation in pixel size and capture distance. These adjustments were used to analyze all ilastik-generated binary images.

### Image processing using machine learning models

#### Annotations and model training.

**Fig 4:**
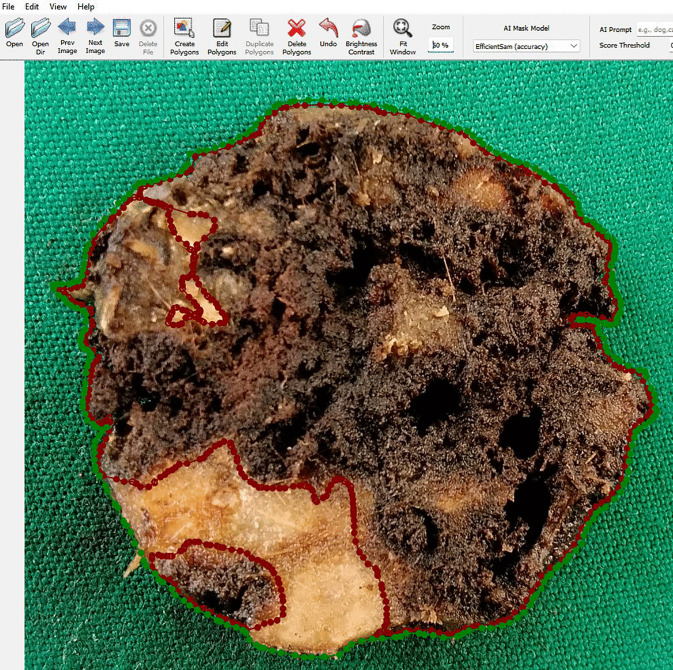
Image annotation using the LabelMe annotation tool. Red polygons delineate damaged corm sections; green polygons delineate corm sections.

Image annotation was completed using LabelMe annotation tool [27] (Fig 4). Irregular polygons were drawn on all images to delineate the boundaries of the entire corm and damaged corm sections. The resultant annotations were exported in Common Objects in Context format (COCO format) [28].

Annotated images were split into train, test, and validation sets with 260, 65, and 72 images, respectively. Four base models were trained, including two convolutional neural network (CNN) architectures (YOLO_v11 and U-Net 512) and two transformer-based models (SegFormer and MiT-b0). Among these, YOLO_v11 is an object detection model that identifies and localizes regions of interest using bounding boxes, thereby estimating object location and spatial extent through rectangular regions that may include non-damaged areas and provide only approximate measurements. In contrast, U-Net 512, SegFormer, and MiT-b0 are semantic segmentation models that assign class labels at the pixel level, enabling precise delineation of damaged tissue and accurate estimation of the proportion of affected area. Although deep learning models generally benefit from large training datasets, these architectures are designed for efficient learning and can perform reliably with moderate dataset sizes when image variability, such as background noise and unrelated features, is minimized. In this study, the use of a uniform background reduced non-biological variation and minimized visual noise, allowing the models to focus on relevant features associated with banana weevil damage.

Each model was hyperparameter-tuned differently, with YOLO_v11 set to batch size 8 and 200 epochs, U-Net 512 set to batch size 2 and 300 epochs, MiT-b0 set to batch size 8 and 40 epochs, and SegFormer set to batch size 8 and 40 epochs. Although YOLO_v11 and U-Net 512 were trained for many epochs, early stopping was used to monitor validation loss, and training was terminated when it stopped decreasing, thereby preventing overfitting. These specific adjustments were made to optimize the training process for the pre-trained models to attain good performance with a small dataset, although most parameters were left as default.

#### Image detection using a trained model.

**Fig 5:**
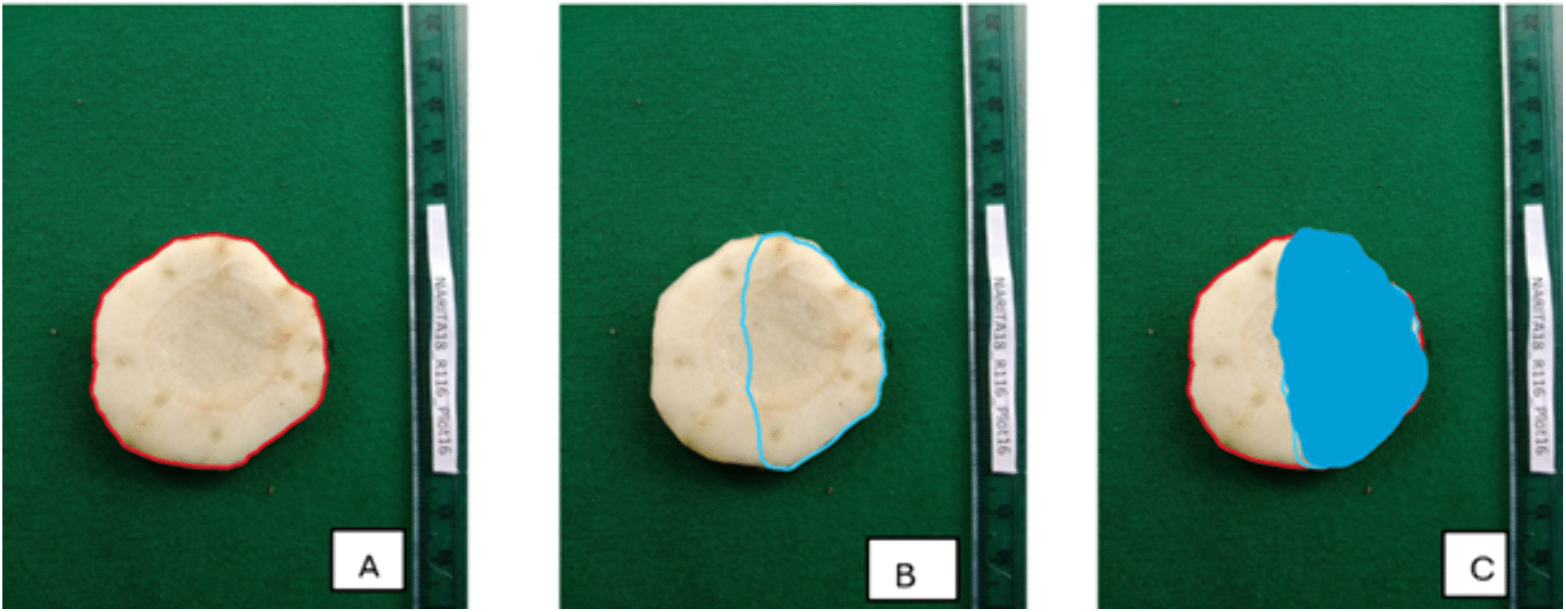
An illustration of IOU.(A)-ground truth region with red boundary; (B)-predicted region with blue boundary; (C)-area of intersection between ground truth and predicted regions (shaded blue) – which is nearly 0.5 IOU since the area of overlap is almost half the ground truth and prediction region (0.2X magnification).

Prediction accuracy for each model was evaluated on the testing dataset of 65 images using image segmentation. Performance was measured using Intersection Over Union (IOU), which measures overlap between predicted area and ground truth as a ratio of area of intersection to area of union between ground truth and predicted regions [29]. IOU ranged from 0 to 1, with higher values indicating better predictions. This metric was used to assess model accuracy when predicting regions in an image belonging to a certain class (Fig 5).

#### Background removal using a trained machine learning model.

**Fig 6:**
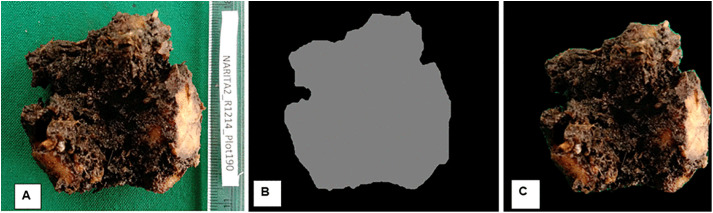
(A)-original input image that is passed through the model; (B)- model predicted mask returned after analysis, and (C)- result of background elimination (0.3x magnification).

A three-step Python script was followed. In step 1, an input image was loaded and passed through the trained machine learning model. In step 2, the model analyzed the image and returned a prediction for the mask representing the corm in the image and in step 3, the predicted mask was combined with the input image to retain only the corm and eliminate the background from the image (Fig 6).

#### Quantification of healthy and damaged sections and model deployment.

After the background removal step, images contained pixels for only the damaged and healthy sections of the corm. These sections were clustered using a pixel-based thresholding technique that used binary classification to determine whether values were larger or less than 120-threshold value, the optimum to distinguish between damaged and healthy pixels in the corm [26]. The input images were converted to grayscale and a blurring transformation to improve the detection of different sections. Two image detection thresholds were used to identify damaged sections in the grayscale images. First, the damaged sections were segmented as white and healthy sections as black to obtain damaged section pixels. In the second step, the healthy section was segmented as white and the damaged section as black to obtain healthy section pixels. The healthy and damaged pixels were summed up to obtain the entire corm pixels and percentage damage calculated following Equation 1 below.


$$\begin{array}{c} \text{Damage Percentage }=\;\frac{damage\;pixels}{cormpixels}\times \text{100} \end{array}$$ (1)

The Python script developed was deployed to automatically analyze all images in the dataset, producing results as comma-separated values (csv) in an Excel file format.

### Data analysis

#### Statistical analysis.

The Pandas python library was used to compute descriptive statistics, including observed means, standard deviations, and mean difference (bias value) [30,31]. Observed means refer to arithmetic means calculated directly from raw data. The observed mean of each score method, the bias value or mean difference, and the standard deviation were calculated as described in S1 Text.

#### Assessing agreement among score methods.

A Bland-Altman plot was generated using the Python library Matplotlib to visualize and assess agreement among the six methods [32]. The upper and lower limits of agreement (LoA) were set at ±1.96 standard deviations to determine the range within which differences among the methods were expected to lie for 95% of observations from the mean difference [33]. The precision of the Bland-Altman LoA was measured in terms of approximate interval width, which is twice the value of the margin of error or the difference between the upper and lower bounds of the confidence interval [33]. The limits of agreement (LoA) were computed as described in S1 Text. The correlation coefficient was calculated using the NumPy library, while Lin’s concordance correlation coefficient (CCC) was computed using the Statsmodels Python library as described in S1 Text [34]. Lin’s CCC measures both precision and accuracy and ranges from 0 to +1.

Interpretation of Lin’s CCC according to [35] (Table 1).

**Table 1:** Interpretation of Lin’s CCC values.

Value of the Lin’s CCC	Interpretation
>0.99	Almost perfect
0.95 to 0.99	Substantial
0.90 to 0.95	Moderate
<0.9	Poor

Matplotlib library was used to plot concordance correlation coefficient scatter plot [32].

Differences in percentage damage among genotypes and scoring methods, as well as their interaction, were then evaluated using a linear mixed-effects model. The model was fitted by restricted maximum likelihood (REML) in GenStat (24th version) [36], accounting for the partially replicated design and repeated assessment of the same corm samples across methods. Fixed effects included genotype, scoring method, and their interaction (genotype × method), while experimental block and individual corm samples were treated as random effects to account for the hierarchical design and non-independence of repeated measurements following the linear mixed model below. Model assumptions were assessed using diagnostic plots of REML residuals. These included quantile-quantile (Q-Q) plots to evaluate normality and residual versus fitted value plots to assess homoscedasticity and linearity. The residuals showed no substantial deviation from normality, indicating that the model assumptions were adequately satisfied.


$$\begin{array}{c} Y_{ijk}=\mu +G_{i}+M_{j}+(GM{)}_{ij}+B_{k}+S_{I(j)}+e_{ijkl} \end{array}$$

where *Y*_*ijk*_ is the damage score, μ is the grand mean, *G*_*i*_ is the fixed effect of genotype, *M*_*j*_ is the fixed effect of damage scoring method, *(GM)*_*ij*_ is the fixed interaction between genotype and damage scoring method, *B*_*k*_ is the random effect of block, *S*_*I*_ is the random effect of sample (corm) nested within genotypes, and *e*_*ijkl*_ is the residual error. Following significant differences in genotype, and weevil scoring method, pairwise comparisons among adjusted means were conducted using Fisher’s protected least significant difference (LSD) test at α = 0.05 to identify which means differed significantly [37].

## Results

### Performance of object detection and segmentation models

The performance of the object detection model (YOLO_v11), which identifies and localizes damage using bounding boxes, was evaluated using mean Average Precision (mAP), a standard metric for object detection. While bounding boxes are suitable for counting or locating affected regions, they do not provide pixel-level information. On the other hand, segmentation models, including U-Net 512, MiT-B0, and SegFormer, which classify each pixel, were assessed using Intersection Over Union (IoU), the primary metric for semantic segmentation, which quantifies pixel-wise overlap between predicted and ground truth masks. YOLO_v11 attained a mAP score of 0.909 for corm detection and 0.611 for damage detection. The segmentation models demonstrated strong performance across all evaluated classes, ranging from 0.839 to 0.876 for the corm class and 0.892 to 0.899 for the damage class (Table 2).

**Table 2:** Performance metrics for object detection and segmentation models.

Task	Model	Metric	Corm class	Damage class	All classes
Object detection	YOLO_v11	mAP	0.909	0.611	0.760
	U-Net 512	IoU	0.875	0.899	0.923
Sematic segmentation	MiT-b0	IoU	0.876	0.892	0.921
	SegFormer	IoU	0.839	0.894	0.909

### Training dynamics and generalization of the models

**Fig 7:**
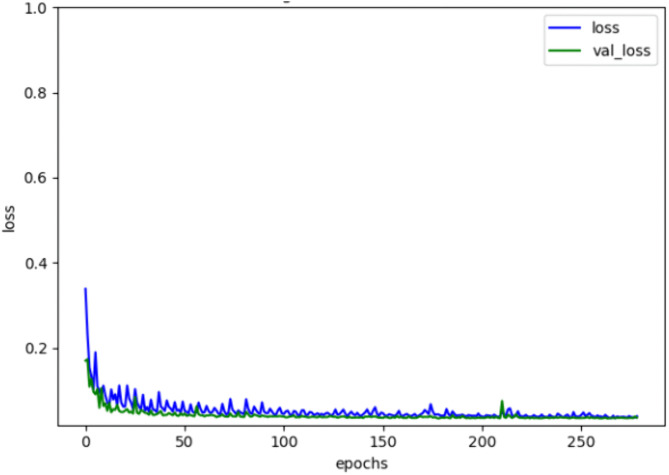
Training and validation loss curves for the U-Net 512 model across epochs, illustrating the model’s optimization behavior and generalization performance during training.

Both training (blue) and validation (green) loss curves for U-Net 512 model decreased sharply during the initial epochs and stabilized by approximately epoch 50, indicating rapid initial learning. The two curves closely overlapped throughout the 250 epochs, with only minor fluctuations that diminished over time (Fig 7). No major divergence was observed, indicating good generalization.

**Fig 8:**
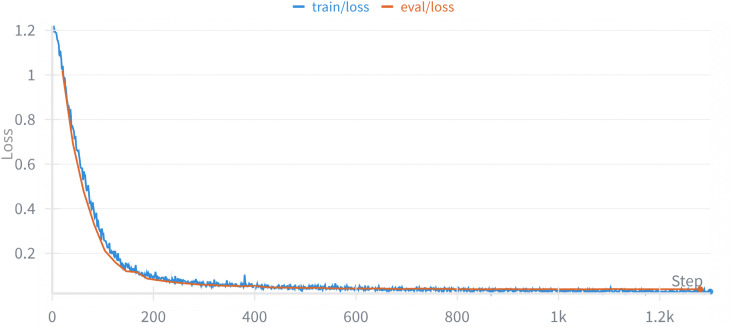
Training and validation loss curves for SegFormer model showing optimization behavior and generalization performance during training.

Training (blue) and validation (orange) loss curves for SegFormer model declined rapidly within the first 200 steps. After that, the loss continued to decrease slowly until it leveled off at a low and stable value (Fig 8). The curves remained closely aligned across all training steps, with no widening gap between training and validation loss. Training and validation loss values levelled out and overlapped in later stages, indicating effective optimization and stable generalization.

**Fig 9:**
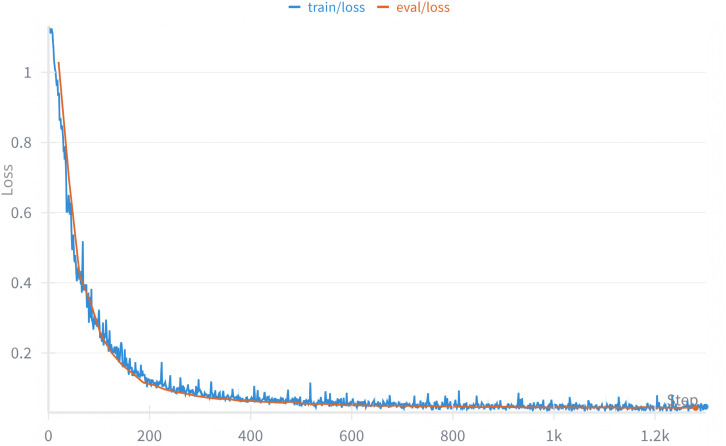
Training and validation loss curves across training steps for MiT-b0 model showing convergence behavior and generalization trends.

Training and validation loss curves for MiT-b0 model decreased significantly during the first 300 steps, followed by a slow and steady drop toward very low loss values. Validation loss consistently tracked training loss throughout, indicating stable learning and good generalization despite minor fluctuations (Fig 9).

**Fig 10:**
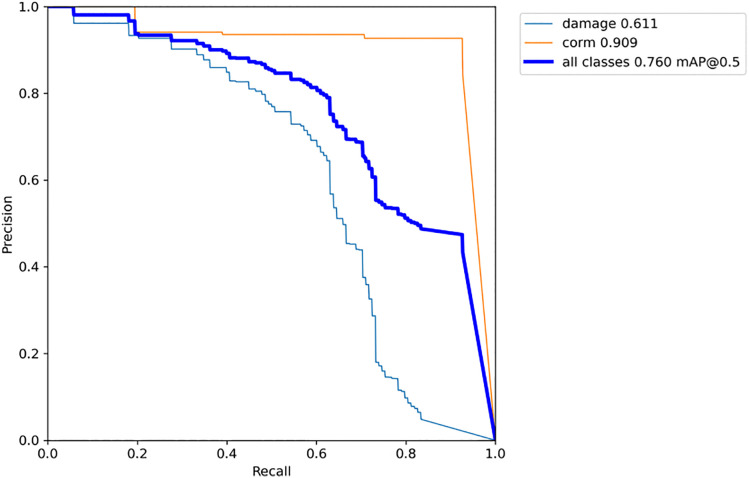
Precision-Recall curve for YOLO_v11 model.

The precision-recall (PR) curve of the trained YOLO_v11 in detecting damage and corm classes is shown in Fig 10. The average precision of corm feature was 0.909 compared to the damage features at 0.611. The mean average precision (mAP) was 0.760 at an intersection-over-union (IoU) threshold of 0.5. A steep decline in the “damage” curve at higher recall levels was also observed.

### Comparing visual observation and image analysis

#### Observed mean and standard deviation scores.

The highest observed means were obtained for visual observation and YOLO_v11, and the lowest for ImageJ. The observed means for all the other machine learning models fell within a similar range, intermediate between visual observation and ImageJ. Conversely, ImageJ had the lowest standard deviation, while visual observation had the highest (Table 3). This indicated that ImageJ scores were closest to the observed mean, whereas visual observation scores deviated more from the observed mean compared with the other methods.

**Table 3:** Comparison of observed mean and standard deviation (SD) scores between visual observation and image analysis.

Score method	Observed mean ± SD (%)
Visual observation	45.15 ± 28.22
ImageJ	39.63 ± 23.92
MiT-b0	42.39 ± 27.27
SegFormer	42.29 ± 27.11
U-Net 512	42.42 ± 27.00
YOLO_v11	46.26 ± 27.67

#### Assessing agreement between visual observation and image analysis.

**Fig 11:**
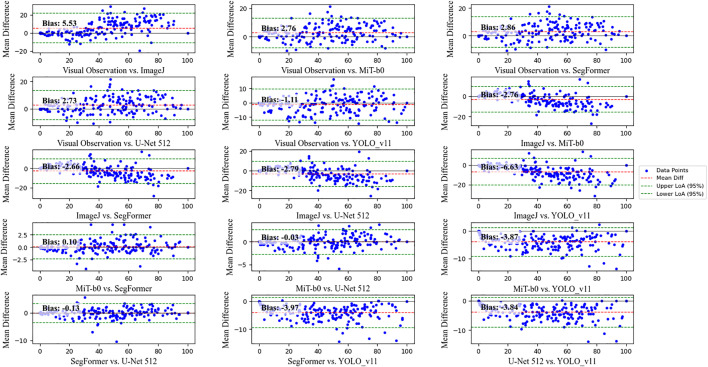
Bland-Altman plot of mean differences among weevil damage assessment methods.

From the Bland-Altman plot in Fig 11, we observed very low bias values ranging from 0.03 to 0.13 among machine learning models when compared with each other, except for YOLO_v11, which were moderately higher, ranging from 3.84 to 3.97 when compared with the other models. In contrast, visual observation and ImageJ showed moderately higher bias, ranging from 0.11 to 5.53 and 2.66 to 6.63, respectively, when compared with machine learning methods (Fig 11). The Bland-Altman plots showed a narrower width of agreement for machine learning models, suggesting consistency and lower variability in differences compared to visual observation and ImageJ, which displayed wider agreement intervals when compared with machine learning methods.

#### Concordance Correlation Coefficient (CCC) between paired measurements from different methods on the same sample.

**Fig 12:**
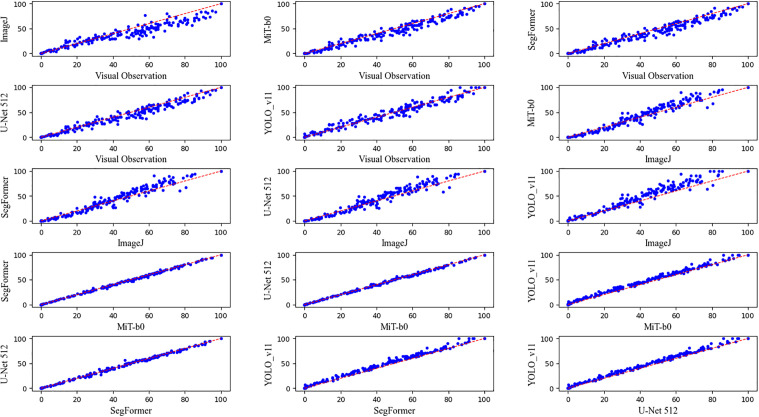
Lin’s concordance correlation coefficient scatter plot among weevil damage assessment methods.

Lin’s CCC scatter plots, comparing damage measurements across machine learning models, visual observation, and ImageJ, showed varying agreement levels among the methods (Fig 12). Pairwise comparisons between machine learning models, with the exception of YOLO_v11 gave a perfect CCC and Pearson’s correlation of 1.00 each, with all data points tightly aligned along the 1:1 line. YOLO_v11 exhibited a substantial agreement with other machine learning models (CCC = 0.99). Visual observation showed substantial agreement with all machine learning methods (CCC = 0.96, Pearson’s r = 0.98). ImageJ also showed moderate agreement with all machine learning models (CCC = 0.94, Pearson’s r = 0.98) except with YOLO_v11 (CCC = 0.90, Pearson’s r = 0.97). However, the comparison between visual observation and ImageJ gave a poor CCC of 0.89, with greater scatter, indicating poor agreement due to variability in manual methods (Table 4).

**Table 4:** Concordance measures among weevil damage scoring methods.

Score methods	Concordance Measures	
	Pearson correlation coefficient (r)	Lin’s CCC
Visual observation vs. ImageJ	0.96	0.89
Visual observation vs. all four machine learning models	0.98	0.96
ImageJ vs. YOLO_v11	0.97	0.90
ImageJ vs. MiT-b0, SegFormer and U-Net 512	0.98	0.94
U-Net 512 vs. MiT-b0	1	1
U-Net 512 vs. SegFormer	1	1
U-Net 512 vs. YOLO_v11	1	0.99
MiT-b0 vs. SegFormer	1	1
MiT-b0 vs. YOLO_v11	1	0.99
SegFormer vs. YOLO_v11	1	0.99

#### Effects of genotypes, scoring method and their interaction during damage scoring.

Table 5 presents the fixed effects evaluated using Wald chi-square statistics for genotype, damage scoring method, and their interaction, based on a REML-fitted linear mixed-effects model. Significant effects were detected for genotype (p < 0.001), scoring method (p < 0.001), and the genotype by method interaction (p < 0.001) (Table 5). Mean separation among genotypes within each scoring method was performed using Fisher’s protected least significant difference test [37] (S2 Table). Genotype resistance rankings varied across scoring methods (Table 6). Mean comparisons among scoring methods revealed significant differences between ImageJ, visual observation, and YOLO_v11(Table 7). In contrast, no significant differences were observed among MiT-b0, SegFormer, and U-Net 512. However, these three models differed significantly from ImageJ, visual observation, and YOLO_v11. Additionally, visual observation and YOLO_v11 also differed significantly from each other and from all other methods (Table 7).

**Table 5:** Wald chi-square tests used to evaluate the significance of the fixed effects from linear mixed effect model by REML.

Fixed effect	d.f	Wald X^2^	P-value
Genotype	21	72.97	<0.001
Weevil scoring method	2	420.53	<0.001
Weevil scoring method*Genotype	105	170.96	<0.001

d.f = Degrees of freedom; Wald X^2^ = Wald chi-square

**Table 6:** Statistical comparison of genotype performance relative to the resistant check Calcutta 4 and susceptible check Mbwazirume across different damage scoring methods using Fisher’s protected least significant difference (LSD) test (α = 0.05).

Genotype	Visual observation			YOLO_v11			MIT-b0			U-Net 512			SegFormer			ImageJ		
	P(C4)	P(Mb)	res	P(C4)	P(Mb)	res	P(C4)	P(Mb)	res	P(C4)	P(Mb)	res	P(C4)	P(Mb)	res	P(C4)	P(Mb)	res
Calcutta 4		0.001	R		0.001	R		0.001	R		0.001	R		0.001	R		0.002	R
NARITA 21	0.289	0.040	R	0.325	0.029	R	0.336	0.038	R	0.331	0.030	R	0.339	0.039	R	0.319	0.063	R
NARITA 23	0.240	0.053	I	0.277	0.037	R	0.301	0.045	R	0.325	0.030	R	0.307	0.046	R	0.237	0.094	I
NARITA 17	0.388	0.057	R	0.232	0.048	R	0.277	0.051	I	0.252	0.050	R	0.264	0.057	I	0.227	0.099	I
NARITA 10	0.030	0.350	S	0.016	0.451	S	0.016	0.542	S	0.010	0.535	S	0.014	0.586	S	0.029	0.503	S
NARITA 16	0.012	0.559	S	0.004	0.782	S	0.007	0.756	S	0.006	0.709	S	0.006	0.819	S	0.021	0.598	S
NARITA 11	0.016	0.564	S	0.009	0.669	S	0.008	0.802	S	0.007	0.772	S	0.010	0.756	S	0.021	0.671	S
Mchare	0.002	0.719	S	0.001	0.754	S	0.002	0.816	S	0.001	0.845	S	0.002	0.778	S	0.004	0.747	S
NARITA 7	0.004	0.864	S	0.005	0.711	S	0.004	0.872	S	0.005	0.799	S	0.004	0.910	S	0.006	0.944	S
Mbwazirume	0.001		S	0.001		S	0.001		S	0.001		S	0.001		S	0.002		S
NARITA 19	0.004	0.849	S	0.003	0.851	S	0.002	0.978	S	0.003	0.904	S	0.002	0.918	S	0.003	0.864	S
NARITA 26	0.005	0.788	S	0.004	0.813	S	0.004	0.875	S	0.005	0.770	S	0.004	0.899	S	0.003	0.858	S
NARITA 18	0.002	0.944	S	0.001	0.734	S	0.001	0.700	S	0.001	0.756	S	0.001	0.666	S	0.001	0.700	S
NARITA 2	0.001	0.817	S	0.001	0.681	S	0.001	0.627	S	0.001	0.699	S	0.001	0.540	S	0.001	0.622	S
NARITA 13	0.001	0.778	S	0.001	0.686	S	0.001	0.595	S	0.001	0.671	S	0.001	0.569	S	0.001	0.603	S
NARITA 1	0.0003	0.564	S	0.001	0.524	S	0.001	0.425	S	0.001	0.448	S	0.001	0.409	S	0.001	0.595	S
NARITA 4	0.0001	0.206	S	0.0001	0.299	S	0.0001	0.202	S	0.0001	0.223	S	0.0001	0.194	S	0.0001	0.188	S
NARITA 15	0.0001	0.100	S	0.0001	0.176	S	0.0001	0.110	S	0.0001	0.121	S	0.0001	0.095	S	0.0001	0.158	S
Obino l’Eewai	0.0001	0.356	S	0.0001	0.363	S	0.0001	0.284	S	0.0001	0.297	S	0.0001	0.272	S	0.0001	0.108	S
NARITA 12	0.0001	0.201	S	0.001	0.173	S	0.0001	0.104	S	0.0001	0.125	S	0.0001	0.091	S	0.0001	0.082	S
NARITA 9	0.0001	0.051	S	0.0001	0.065	S	0.0001	0.035	MS	0.0001	0.050	MS	0.0001	0.032	MS	0.0001	0.025	MS
NARITA 14	0.0001	0.086	S	0.0001	0.112	S	0.0001	0.061	S	0.0001	0.078	S	0.0001	0.052	S	0.0001	0.018	MS

Abbreviations: P(C4), p-value indicating whether a genotype differs significantly from the resistant check Calcutta 4; P(Mb), p-value indicating whether a genotype differs significantly from the susceptible check Mbwazirume; res, genotype response category; S, susceptible; R, resistant; I, intermediate; MS, more susceptible than the susceptible check Mbwazirume.

**Table 7:** Mean separation of scoring methods means using Fisher’s protected least significant difference (LSD) test at α = 0.05. Means followed by the same letter within columns are not significantly different.

Method	Adjusted mean ± SE
ImageJ	40.58 ± 1.91 a
SegFormer	43.25 ± 1.91 b
MiT-b0	43.34 ± 1.91 b
U-Net 512	43.38 ± 1.91 b
Visual observation	46.11 ± 1.91 c
YOLO_v11	47.21 ± 1.91 d

SE = Standard error.

We further compared the costs and benefits of the different scoring methods under operational conditions at the International Institute of Tropical Agriculture (IITA) in Uganda, where the study was conducted. Cost estimates were derived from operational expenses at IITA and are reported in United States dollars (USD) (Table 8). The comparison was across key parameters highlighting trade-offs and suitability for various applications. While visual observation presented as a low-cost option for tools, it is labor-intensive, time-consuming, and less reliable, with notable limitations in scalability, flexibility, and efficiency. On the other hand, ImageJ provided a balanced approach, offering moderate costs, improved performance, and requiring moderate labor. Machine learning models demonstrated the highest performance, particularly in scalability, flexibility, and processing speed, processing samples in just two seconds with very high reliability. Despite higher data storage requirements, machine learning is best suited for large-scale and dynamic applications, while ImageJ offers a middle ground between cost and performance.

**Table 8:** Comparison of resource requirements and operational considerations for Visual observation, ImageJ, and Machine learning in banana weevil damage assessment.

Component	Cost Element	Visual observation	Image J	Machine learning models
**Tools**	Camera purchase (USD)	_	272	272
	Computer (USD)	_	600	600
	Accessories/Consumables (USD)	14	50	50
**Operations**	Labor	High	Moderate	Moderate
	Training	High	High	High
	Fieldwork	High	High	High
**Data capture**	Time per sample (sec)	60	30	1–2
	Cost per sample	High	Moderate	Moderate
	Data storage	Low	High	High
	Error/Reprocessing costs	High	Low	Low
**Other considerations**	Contingencies	High	Low	Low
	Scalability	Limited	High	Very high
	Flexibility	Limited	High	Very high
	Reliability	Low	High	Highly

## Discussion

Phenotyping for resistance to banana weevil is currently achieved through expert estimation [24]. This approach is slow and subject to personal bias, impacting data quality, processing speed, and reproducibility. To address these limitations ImageJ and machine learning models were evaluated. Among the evaluated models, transformer-based architectures MiT-b0 and SegFormer attained higher intersection-over-union score, attributed to the use of self-attention mechanisms, which enabled the capture of long-range dependencies and global context, thereby increasing segmentation precision at the pixel level [38–40]. However, the performance of U-Net 512, the CNN-based model, was also similar to that of transformer models. It outperformed YOLO_v11, another CNN-based model primarily due to encoder-decoder architecture with skip connections which allows effective combination of local details with global contextual information [38]. The deviation of YOLO_v11 performance from other models is attributed to its design as an object detection model adapted for segmentation, making it less precise for pixel-wise tasks [41]. This limitation of YOLO_v11 was evidenced across the 370 corm samples during deployment were it underperformed compared to MiT-b0, SegFormer, and U-Net 512. Notably, MiT-b0, SegFormer, and U-Net 512 agreed perfectly on damage score values per corm sample, as evidenced by their Lin’s concordance correlation coefficient values and the corresponding Lin’s CCC scatter plots, which showed complete alignment. This suggests they generated nearly identical segmentation masks for the corm images.

Visual observation showed a significant difference in adjusted mean compared with MiT-b0, U-Net 512, and SegFormer, yet maintained substantial agreement. This indicated that, although visual observation produced higher damage score values on average, its measurements closely aligned with those of MiT-b0, U-Net 512, and SegFormer models at the individual corm sample level. The significant difference in mean values is attributed to the subjective nature of visual observation compared to machine learning models.

ImageJ also differed significantly in adjusted mean with MiT-b0, U-Net 512, SegFormer and YOLO_v11, while demonstrating moderate agreement according to Lin’s CCC values. This indicated that ImageJ’s scores were modestly less consistent compared to machine learning at the individual corm sample level. The difference is attributed to ImageJ’s reliance on manual thresholding, which limited its ability to capture more complex patterns in corm images, whereas machine learning models were better suited to handling such complex features.

Nonetheless, Lin’s CCC scatter and Bland-Altman plots comparing visual observation and ImageJ with machine learning models showed proportional bias, suggesting an imperfect linear relationship between the methods [34,42], with agreement varying by damage severity. Proportional bias probably arose due to one method over- or underestimating relative to another as the true values increased or decreased [33,43]. Visual observation aligned more closely with image analysis at lower damage scores but tended to diverge at higher scores, likely due to measurement error especially when portions of the corm were lost to extensive weevil damage, yet ImageJ and machine learning do not capture this change in corm shape.

A significant genotype effect was observed, indicating differences in weevil resistance among the genotypes. However, the significant interaction between genotype and weevil damage scoring method suggested that genotype rankings and the extent of observed damage per genotype varied across methods. This was evident from the mean separation of genotypes per scoring method, were some genotypes ranked differently in terms of resistance depending on the method used, highlighting that the choice of scoring method can influence the magnitude of damage quantified.

We thus recommend adopting machine learning methods to minimize bias in scoring weevil damage, especially when weevil assessment is completed by different people. Aside from the need to capture quality images and train the software to distinguish between damaged and healthy tissue, machine learning significantly reduces the time required to analyze large datasets accurately with a single command. In addition to high precision and accuracy, machine learning processes images every two seconds compared to 30 seconds for ImageJ and approximately 1 minute for visual observation. The images and results from machine learning, stored on MusaBase, can support the development of a detailed database; however, analysis is constrained by the requirement for quality images. This limitation can be addressed by using DigiEye, an advanced imaging system that captures high-resolution images under controlled light conditions. We piloted the use of DigiEye, however, the data was not sufficient to include in this publication. DigiEye represents a significant advancement in image analysis technology, offering precision, consistency, and objectivity [44]. Despite limitations in terms of cost and technical requirements, the benefits make it a valuable tool in corm damage scoring. In addition to capturing the image, it can be used to convert the image into binary reducing the initial steps for converting images to binary using ilastik when using ImageJ. Ongoing work aims to extend machine learning approaches to phenotyping resistance to *Fusarium oxysporum* f. sp. *cubense* race 1 (FOC-R1) and *Radopholus similis* in banana. Similar image-based methodologies are also being adopted by the International Institute of Tropical Agriculture’s cassava breeding programs to quantify tuber damage caused by cassava brown streak disease, demonstrating the adaptability of Artificial intelligence-driven phenotyping across crops. In Uganda, such applications have the potential to support rapid, objective, and scalable screening for resistance to major pests and diseases, thereby improving decision-making during genotype selection in crop improvement programs.

## Supporting information

S1 Fig Banana plants established in buckets. Banana plants were established in buckets and covered with weevil-proof nets to prevent adult weevils from escaping after infestation of the experiment.
(TIFF)

S1 Table Experimental layout of the partially replicated design. Layout of the partially replicated experimental design with three blocks used to evaluate genotype response to weevil infestation.
(XLSX)

S2 Table Fisher’s protected least significant difference (LSD) for each scoring method. Results of Fisher’s protected least significant difference test showing genotype mean separation within each scoring method. The test was performed to identify genotypes that differed significantly from each other and theirs resistance performance.
(XLSX)

S1 Text Statistical equations used to calculate observed mean, bias values, standard deviation, limits of agreement (LoA), Correlation coefficient and Lin’s concordance correlation coefficient (CCC). (DOCX)

## References

[1] Kiggundu A, Gold CS, Vuylsteke D. Response of banana cultivars to banana weevil attack. Uganda J Agric Sci. 2000;5(2):36–40.

[2] Gold CS, Pena JE, Karamura EB. Biology and integrated pest management for the banana weevil Cosmopolites sordidus (Germar) (Coleoptera: Curculionidae). Integr Pest Manag Rev. 2001;6(2):79–155. doi: 10.1023/a:1023330900707

[3] Viljoen A, Mahuku G, Massawe C, Ssali RT, Kimunye J, Mostert G, et al. Banana pests and diseases: field guide for disease diagnostics and data collection. Ibadan, Nigeria: International Institute of Tropical Agriculture; 2016.

[4] Rukazambuga NDTM, Gold CS, Gowen SR. Yield loss in East African highland banana (Musa spp., AAA-EA group) caused by the banana weevil, Cosmopolites sordidus Germar. Crop Protec. 1998;17(7):581–9. doi: 10.1016/S0261-2194(98)00056-1

[5] Gold CS, Kagezi GH, Night G, Ragama PE. The effects of banana weevil, Cosmopolites sordidus, damage on highland banana growth, yield and stand duration in Uganda. Ann Appl Biol. 2004;145(3):263–9. doi: 10.1111/j.1744-7348.2004.tb00382.x

[6] Ortiz R, Vuylsteke D, Dumpe B, Ferris RSB. Banana weevil resistance and corm hardness in Musa germplasm. Euphytica. 1995;86(2):95–102. doi: 10.1007/bf00022014

[7] Li W, Deng Y, Ning Y, He Z, Wang G-L. Exploiting broad-spectrum disease resistance in crops: from molecular dissection to breeding. Annu Rev Plant Biol. 2020;71:575–603. doi: 10.1146/annurev-arplant-010720-022215 32197052

[8] Elliott K, Berry JC, Kim H, Bart RS. A comparison of ImageJ and machine learning based image analysis methods to measure cassava bacterial blight disease severity. Plant Methods. 2022;18(1):86. doi: 10.1186/s13007-022-00906-x 35729628 PMC9210806

[9] Abramoff MD, Magalhães PJ, Ram SJ. Image processing with ImageJ. Biophotonics Int. 2004;11(7):36–42.

[10] Mutka AM, Bart RS. Image-based phenotyping of plant disease symptoms. Front Plant Sci. 2015;5:734. doi: 10.3389/fpls.2014.00734 25601871 PMC4283508

[11] Laflamme B, Middleton M, Lo T, Desveaux D, Guttman DS. Image-based quantification of plant immunity and disease. Mol Plant Microbe Interact. 2016;29(12):919–24. doi: 10.1094/MPMI-07-16-0129-TA 27996374

[12] Guiet R, Burri O, Seitz A. Open-source tools for biological image analysis. In: Rebollo E, Bosch M, editors. Computer optimized microscopy: methods and protocols. New York, NY: Springer; 2019. pp. 23–37. doi: 10.1007/978-1-4939-9686-5_2 31432473

[13] Stawarczyk M, Stawarczyk K. Use of the ImageJ program to assess the damage of plants by snails. Chem Didact Ecol Metrol. 2015;20(1–2):67–73. doi: 10.1515/cdem-2015-0007

[14] Agehara S. Simple imaging techniques for plant growth assessment. EDIS. 2020;2020(1):5. doi: 10.32473/edis-hs1353-2020

[15] Kamilaris A, Prenafeta-Boldú FX. Deep learning in agriculture: a survey. Comput Electron Agric. 2018;147:70–90. doi: 10.1016/j.compag.2018.02.016

[16] Ubbens JR, Stavness I. Deep plant phenomics: a deep learning platform for complex plant phenotyping tasks. Front Plant Sci. 2017;8:1190. doi: 10.3389/fpls.2017.01190 28736569 PMC5500639

[17] Singh A, Ganapathysubramanian B, Singh AK, Sarkar S. Machine learning for high-throughput stress phenotyping in plants. Trends Plant Sci. 2016;21(2):110–24. doi: 10.1016/j.tplants.2015.10.015 26651918

[18] Lindow SE. Quantification of foliar plant disease symptoms by microcomputer-digitized video image analysis. Phytopathology. 1983;73(4):520. doi: 10.1094/phyto-73-520

[19] Arnal Barbedo JG. Digital image processing techniques for detecting, quantifying, and classifying plant diseases. SpringerPlus. 2013;2(1):660. 24349961 10.1186/2193-1801-2-660 PMC3863396

[20] Veerendra G, Swaroop R, Dattu DS, Jyothi ChA, Singh MK. Detecting plant diseases, quantifying and classifying digital image processing techniques. Mater Today: Proc. 2022;51:837–41. doi: 10.1016/j.matpr.2021.06.271

[21] Williams ER, John JA, Whitaker D. Construction of more flexible and efficient P-rep designs. Aust N Z J Stat. 2014;56(1):89–96. doi: 10.1111/anzs.12068

[22] VSN International. CycDesigN version 8.0: Optimal experimental design software. Hemel Hempstead, UK: VSN International; 2013. Available from: https://vsni.co.uk/software/cycdesign

[23] Longoria A. Diferencias sexuales en la morfologia externa de Cosmopolites sordidus Germar (Coleoptera: Curculionidae). Ciencias Biol. 1968;1(1):1–11.

[24] Gold CS, Speijer PR, Karamura EB, Tushemereirwe WK, Kashaija IN. Survey methodologies for banana weevil and nematode damage assessment in Uganda. Afr Crop Sci J. 1994;2:309–21.

[25] Konrad D. rembg: remove image background [Internet]. GitHub. [cited 2026 Feb 13]. Available from: https://github.com/danielgatis/rembg

[26] Gonzalez RC, Woods RE. Digital image processing. 4th ed. New York: Pearson; 2018.

[27] Russell BC, Torralba A, Murphy KP, Freeman WT. LabelMe: a database and web-based tool for image annotation. Int J Comput Vis. 2007;77(1–3):157–73. doi: 10.1007/s11263-007-0090-8

[28] Lin TY, Maire M, Belongie S, Hays J, Perona P, Ramanan D, et al. Microsoft COCO: common objects in context. In: Fleet D, Pajdla T, Schiele B, Tuytelaars T, editors. Computer Vision – ECCV 2014. Cham: Springer; 2014. pp. 740–55. doi: 10.1007/978-3-319-10602-1_48

[29] Everingham M, Van Gool L, Williams CKI, Winn J, Zisserman A. The PASCAL visual object classes (VOC) challenge. Int J Comput Vis. 2010;88(2):303–38. doi: 10.1007/s11263-009-0275-4

[30] McKinney W. Pandas: a foundational Python library for data analysis and statistics. Python for High Performance and Scientific Computing. 2011;14(9):1–9.

[31] McKinney W. Python for data analysis: data wrangling with Pandas, NumPy, and IPython. Sebastopol, CA: O’Reilly Media, Inc.; 2012.

[32] Hunter JD. Matplotlib: a 2D graphics environment. Comput Sci Eng. 2007;9(3):90–5. doi: 10.1109/MCSE.2007.55

[33] Bland JM, Altman DG. Statistical methods for assessing agreement between two methods of clinical measurement. Lancet. 1986;1(8476):307–10. doi: 10.1016/s0140-6736(86)90837-8 2868172

[34] Lin LI. A concordance correlation coefficient to evaluate reproducibility. Biometrics. 1989;45(1):255–68. doi: 10.2307/2532051 2720055

[35] McBride GB. A proposal for strength-of-agreement criteria for Lin’s concordance correlation coefficient. Hamilton, New Zealand: National Institute of Water & Atmospheric Research Ltd.; 2005.

[36] GenStat for Windows 24th Edition. Hemel Hempstead, UK: VSN International. https://vsni.co.uk/software/genstat/

[37] Williams LJ, Abdi H. Fisher’s least significant difference (LSD) test. In: Salkind N, editor. Encyclopedia of research design. Sage Publications; 2010. pp. 491–3. doi: 10.4135/9781412961288.n154

[38] Ronneberger O, Fischer P, Brox T. U-Net: convolutional networks for biomedical image segmentation. In: Navab N, Hornegger J, Wells WM, Frangi AF, editors. Medical image computing and computer-assisted intervention – MICCAI 2015. Cham: Springer; 2015. pp. 234–41. doi: 10.1007/978-3-319-24574-4_28

[39] Xie E, Wang W, Yu Z, Anandkumar A, Alvarez JM, Luo P. SegFormer: Simple and efficient design for semantic segmentation with transformers. Adv Neural Inform Process Syst. 2021.

[40] Dosovitskiy A, Beyer L, Kolesnikov A, et al. An image is worth 16 × 16 words: Transformers for image recognition at scale. International Conference on Learning Representations (ICLR). 2021.

[41] Ultralytics. YOLO11: the latest evolution in real-time object detection. Ultralytics Blog. 2024. Available from: https://www.ultralytics.com/blog/yolo11

[42] Chiang TY, Huang CS, Lin J, Chen YH. A tutorial on the concordance correlation coefficient. Taiwan J Public Health. 2016;35(1):TBD.

[43] Hazra A, Gogtay N. Biostatistics series module 6: correlation and linear regression. Indian J Dermatol. 2016;61(6):593–601. doi: 10.4103/0019-5154.193662 27904175 PMC5122272

[44] Kumah C, Zhang N, Raji RK, Pan R. Color measurement of segmented printed fabric patterns in Lab color space from RGB digital images. J Text Sci Technol. 2019;5(1):1–18. doi: 10.4236/jtst.2019.51001

