# Supplementary figures and images for: Digital assessment of banana (Musa spp.) genotype resistance to banana weevil (Cosmopolites sordidus) compared with expert visual assessment
Source: PLoS One. 2026 Jun 29;21(6):e0352433. doi: 10.1371/journal.pone.0352433 (PMC13313373; doi:10.1371/journal.pone.0352433)

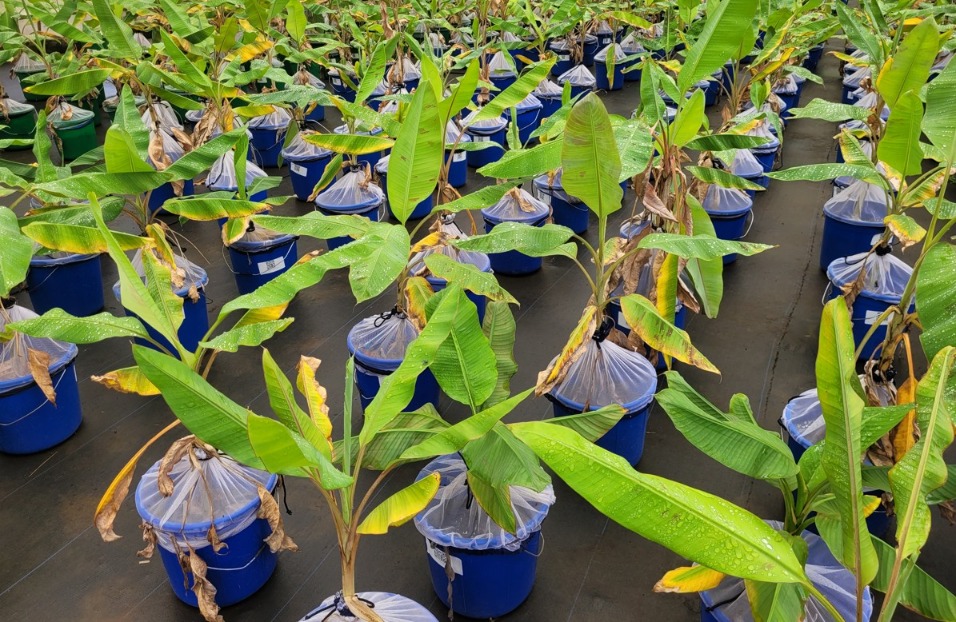

Supplement: S1 Fig — Banana plants were established in buckets and covered with weevil-proof nets to prevent adult weevils from escaping after infestation of the experiment. (TIFF) [file pone.0352433.s001.tiff]
