# Supplementary material for: Digital assessment of banana (Musa spp.) genotype resistance to banana weevil (Cosmopolites sordidus) compared with expert visual assessment
Source: PLoS One. 2026 Jun 29;21(6):e0352433. doi: 10.1371/journal.pone.0352433 (PMC13313373; doi:10.1371/journal.pone.0352433)
Supplement: S1 Text — (DOCX) [file pone.0352433.s004.docx]

**Equation 1. Calculation of the mean of each score method**

Mean score of method =$\boldsymbol{\sum(}Weevil damage values)/(Number of data points\boldsymbol{)}$

Where ∑ (Weevil damage values) represents the sum of all values in a particular weevil damage scoring method, either the visual or image analysis methods.

**Equation 2. Calculating the bias values or mean differences**

Mean difference (Bias) = $\boldsymbol{mean of method A - mean of method B}$

Where method A and B are either visual score or image analysis methods.

**Equation 3. Computing the standard deviation**

Standard deviation = $\frac{\sqrt{\left( \Sigma_{\left( \boldsymbol{i=1} \right)}^{\boldsymbol{n}}\left( \boldsymbol{x}_{\boldsymbol{i}}\boldsymbol{-}\bar{\boldsymbol{x}} \right)^{\boldsymbol{2}} \right)}}{\left( \boldsymbol{n-1} \right)}$

Where ∑ = summation, n = number of data points in the sample, x_i_ = represents each data point, and x̄ = the sample mean.

**Equation 4. Calculation of limits of agreement (LoA)**

Upper limit of agreement (Upper LoA) =$Bias + \left( 1.96 * SD\_diff \right)$

Lower limit of agreement (Lower LoA) =$Bias - (1.96 * SD\_diff)$

Where bias represents the mean difference between two methods, and *SD_diff* is the Standard deviation of the difference

**Equation 5. Calculating the correlation coefficient**

Correlation coefficient = $\frac{\Sigma\left( \boldsymbol{x-}\bar{\boldsymbol{x}} \right)\left( \boldsymbol{y-ȳ} \right)}{\sqrt{\boldsymbol{\Sigma}\left( \boldsymbol{x-}\bar{\boldsymbol{x}} \right)\boldsymbol{2 \Sigma}\left( \boldsymbol{y-ȳ} \right)\boldsymbol{2}}}$

Where X and Y represent values of either visual score or Image analysis score methods; x̅ and ȳ are the means of X and Y, respectively; Σ = the sum of the products and squares over all data points.

**Equation 6. compute Lin’s concordance correlation coefficient (CCC)**

CCC= $\frac{\boldsymbol{(2 * r * \sigma x * \sigma y)}}{\boldsymbol{(\sigma x^2 + \sigma y^2 + (\mu x - \mu y)^2)}}$

Where r = Pearson correlation coefficient; σx and σy are standard deviations of either visual and ImageJ scoring methods, visual and machine learning scoring methods or ImageJ and machine learning scoring methods, respectively; μx and μy are means of either visual and ImageJ scoring methods, visual and machine learning scoring methods, or ImageJ and machine learning scoring methods, respectively.
